# Positive Impact of Mindfulness Meditation on Mental Health of Female Teachers during the COVID-19 Outbreak in Italy

**DOI:** 10.3390/ijerph17186450

**Published:** 2020-09-04

**Authors:** Alessio Matiz, Franco Fabbro, Andrea Paschetto, Damiano Cantone, Anselmo Roberto Paolone, Cristiano Crescentini

**Affiliations:** 1 Department of Languages and Literatures, Communication, Education and Society, University of Udine, 33100 Udine, Italy; franco.fabbro@uniud.it (F.F.); andrea.paschetto@uniud.it (A.P.); anselmo.paolone@uniud.it (A.R.P.); cristiano.crescentini@uniud.it (C.C.); 2 Perceptual Robotics (PERCRO) Laboratory Scuola Superiore Sant’Anna, 56010 Pisa, Italy; 3 Department of Psychology, University of Rome La Sapienza, 00185 Rome, Italy; cantonedamiano@hotmail.com

**Keywords:** COVID-19, longitudinal study, mindfulness meditation, resilience, school teachers, self-reports, women’s mental health

## Abstract

The Covid-19 pandemic and subsequent public health measures were shown to impact negatively on people’s mental health. In particular, women were reported to be at higher risk than men of developing symptoms of stress/anxiety/depression, and resilience was considered a key factor for positive mental health outcomes. In the present study, a sample of Italian female teachers (*n* = 66, age: 51.5 ± 7.9 years) was assessed with self-report instruments one month before and one month after the start of the Covid-19 lockdown: mindfulness skills, empathy, personality profiles, interoceptive awareness, psychological well-being, emotional distress and burnout levels were measured. Meanwhile, they received an 8-week Mindfulness-Oriented Meditation (MOM) course, through two group meetings and six individual video-lessons. Based on baseline personality profiles, analyses of variance were performed in a low-resilience (LR, *n* = 32) and a high-resilience (HR, *n* = 26) group. The LR and HR groups differed at baseline in most of the self-report measures. Pre–post MOM significant improvements were found in both groups in anxiety, depression, affective empathy, emotional exhaustion, psychological well-being, interoceptive awareness, character traits and mindfulness levels. Improvements in depression and psychological well-being were higher in the LR vs. HR group. We conclude that mindfulness-based training can effectively mitigate the psychological negative consequences of the Covid-19 outbreak, helping in particular to restore well-being in the most vulnerable individuals.

## 1. Introduction

The Italian population has recently experienced the first nationwide lockdown of the 21st century due to the Covid-19 infectious disease. After the Covid-19 outbreak in China in December 2019, the epidemic moved to the European region and was declared a pandemic by the WHO on 11 March 2020 [1], when Italy was the worst affected country outside China and was soon becoming the new center of the virus spread. City and regional lockdowns in Italy had already started in February, but the nationwide lockdown started on 9 March 2020, with an estimated 56 million people ordered to remain at home: bars, restaurants, schools and churches were closed and only essential services were permitted (e.g., vital health-care, food stores, electricity/gas/water/fuel suppliers, garbage collection). At the release of the toughest lockdown measures, on 4 May 2020, Italy had officially experienced 210,717 Covid-19 cases and had suffered 28,884 deaths due to this disease [2].

Besides the casualties and the economic loss (with a −9.1% estimated drop in GDP for 2020 [3]) associated with the Covid-19 pandemic, the fear of the virus and the 2-month lockdown had a serious psychological impact on a large part of the Italian population. Early reports emerging in the literature have shown that between 40% and 50% of adults have experienced psychological distress following the Covid-19 outbreak [4,5], and that 30% of adults and children are at high risk for post-traumatic stress symptoms [6]. These results are in line with those previously collected from Chinese samples [7,8,9,10], and extend our previous knowledge of the psychological burden of quarantines coming from past (shorter and smaller scale) experiences of isolation due to public health emergencies and relative confinement measures [11].

Previous experiences with global health emergencies have shown that the effective use of lockdown as a public health approach requires minimizing the harmful effects associated with it: the fundamental steps to this purpose seem to be the ensuring of sufficient primary supplies during the lockdown, and a clear communication, by the authorities, of the reasons for the restrictions and of the protocols to be followed [11], as well as cash transfers and job retention acts [12]. Moreover, other institutional and individual interventions have been proposed to protect mental heath in vulnerable groups, in particular during emergency situations such as the Covid-19 outbreak. These include stigma mitigation [13], psychological/psychiatric assistance [14,15], psychoeducation [16] and teaching self-help methods [15,17].

Teaching evidence-based self-help methods often represents a viable option to address the urgent need of psychological support for large numbers of people, when in-person services are suspended [18,19]. A recent review of the meta-analyses of self-guided interventions to alleviate anxiety, depression and stress concluded that such interventions significantly improve well-being in comparison to control groups, albeit with effect sizes smaller than those associated with traditional guided individual or group therapies [20]. The interventions that proved to be the most effective included self-guided cognitive-behavioral therapy, acceptance and commitment therapy, and Mindfulness-Based Interventions (MBIs).

Mindfulness-Based Interventions are generally based on meditation practices taken from the Buddhist tradition and adapted into contemporary, psychologically-oriented programs [21]. These programs are usually delivered over 8 consecutive weeks, with eight group meetings led by a mindfulness instructor and daily individual practice at home [22]. Several studies have highlighted the benefits of MBIs mainly in terms of self-reported reductions in anxiety, depression and stress levels [23,24,25]. Other improvements due to MBIs have been reported for job burnout [26], interoceptive awareness [27,28], empathy [29,30], character traits [31,32] and psychological well-being [33,34]. It is important to underline that, after the completion of the program, participants can maintain and enhance the benefits gained during the training program by continuing with the meditation on their own. Two decades since the introduction of MBIs, research has showed that they could be effective in promoting well-being also when delivered through a single introductory group meeting, during which the meditation practices are taught and participants carry on the program individually [35,36], or when delivered via the internet [37,38]. MBIs are therefore tools potentially suitable for dealing with stressors arising during lockdown periods, because they can help manage psychological suffering and they can be delivered even in situations where face-to-face meetings are suspended and a large number of people need psychological support.

**Figure 1:**
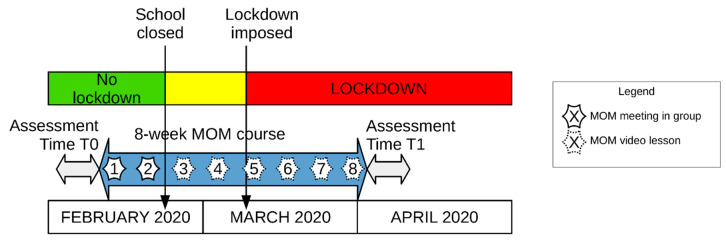
Timeline of Mindfulness-Oriented Meditation (MOM) training delivered to a group of Italian school teachers during the Covid-19 health emergency. The MOM training started with group meetings and continued with individual lessons via the internet.

In the present study, an 8-week Mindfulness-Oriented Meditation course (MOM, which is a standardized MBI) was delivered, through two face-to-face meetings (before these meetings were prohibited by the government due to the spread of the Covid-19 pandemic) and six video-lessons, to a sample of female teachers (Figure 1). Although the MOM course was not originally aimed at mitigating the psychological distress resulting from the Covid-19 pandemic, as this event was unpredictable, it ended up being addressed to a class of the general population most vulnerable to the consequences of this event: many studies have indeed frequently shown that being a woman is one of the risk factors for poor mental health outcomes during the Covid-19 pandemic [5,6,39,40,41,42].

Moreover, it is well known that, when a population faces the same stressful situation, such as in the case of the Covid-19 pandemic, not everyone suffers the same way. Individual adaptive or non-adaptive responses to adverse events depend on a variety of biological, cultural, social and psychological components, which can be encompassed in the concept of psychological resilience [43]. Past research found that resilience is directly related, on the one hand, to secure attachment [44,45], mental health [46,47], psychological well-being [48], empathy [49], mindfulness [50] and interoceptive awareness [51], and, on the other hand, inversely related to childhood trauma [45], anxiety, depression [52] and burnout [53]. Many studies have shown the link between measures of resilience and personality traits, such as harm avoidance, self-directedness, persistence and neuroticism [45,54,55,56,57]. The sample of teachers who received the MBI were studied here in terms of their resilience profile, generated on the basis of temperamental (i.e., mainly innate) and characterological (i.e., mainly due to personal experiences and education) measures taken at baseline with the Temperament and Character Inventory [58,59].

In our study, we therefore analyzed, within the full sample of female teachers receiving the MOM course, the data of a group of teachers with a high-resilience (HR) profile, and the data of a group of teachers with a low-resilience (LR) profile. These teachers were assessed with validated self-report instruments one month before (when the Covid-19 outbreak in China did not seem to threaten the Italian population) and one month after the imposition of the national lockdown in Italy (when the population was shocked by the unprecedented measures taken by the Italian government to deal with the spread of the Covid-19 infectious disease). In this period, the teachers received an 8-week mindfulness meditation course—partly (25%) delivered in person and partly (75%) via the internet. They were assessed in terms of their mindfulness skills, empathy, personality profile, interoceptive awareness, psychological well-being, emotional distress and burnout level.

Three main objectives were pursued in our study. *Objective 1*: to establish whether the two groups of teachers, who had two opposite profiles of resilience at baseline (obtained from teachers’ personality profiles before the MOM course), were different at baseline in the other measures employed in our study as well. Based on previous research on resilience [44,45,46,47,48,49,50,51,52,53,54,55,56,57], we hypothesized that the low-resilience group, compared to the high-resilience group, had worse baseline levels of psychological well-being, mindfulness skills, interoceptive awareness, emotional distress, empathy and burnout (H1).

*Objective 2*: to examine the changes in the two groups that occurred from before to after the MOM course; this also allows observation of the between-group differences after the intervention. Based on the previous literature on the effects of MBIs [23,24,25,26,27,28,29,30,31,32,33,34], which referred to periods free from global health emergencies like that related to the Covid-19 pandemic, we hypothesized that both groups of teachers experienced similar improvements in mindfulness skills, empathy, character traits and interoceptive awareness (H2). As the psychological impact of compulsory isolation in the context of a world-wide pandemic has not previously been examined in relation to mindfulness meditation courses, the analyses of changes in the measures of psychological well-being, burnout and emotional distress (anxiety and depression) were exploratory.

*Objective 3*: to evaluate the teachers’ satisfaction with the MOM course and their perception of the course’s impact on their lives in the context of the Covid-19 outbreak. Based on our many years of experience in conducting MOM courses (see Methods), we hypothesized a moderate to high course satisfaction (H3a). Based on the perceived effectiveness of mindfulness meditation as a self-help method [20], we also hypothesized that participants could perceive mindfulness meditation as a useful practice in living through the Covid-19 lockdown period (H3b).

## 2. Method

### 2.1. Procedure

An 8-week Mindfulness-Oriented Meditation (MOM) training program was organized by the University of Udine on behalf of the Prevention Department and financed by the Friuli Venezia-Giulia region, in the northeast of Italy in a preventive medicine project. This project was originally designed to support teachers’ mental health (i.e., to prevent professional burnout and improve well-being [60]). After participant recruitment, two parallel MOM courses were scheduled and two schools were chosen as the locations for the courses. Participants enrolled in one of the two courses based on the location of the course (the locations of the courses were about 60 km away). The MOM courses were held by two of the authors (A.M., a professional socio-health educator, and A.P., a psychologist), who have more than five years of experience in practicing and teaching mindfulness meditation.

In the original design of the study, data collected from participants in the MOM training should have been compared with data from control participants obtained after the end of the MOM course (in two sessions, eight weeks apart—the same time that elapsed between the pre- and post-MOM course assessments). However, due to the Covid-19 outbreak in Italy in the first weeks of the MOM course, the control group of participants was not assessed; data from teachers attending the MOM course who were assessed one month before and one month after the national lockdown could not have been fairly compared to the data from teachers who were in a condition with fewer restrictions thanks to the loosening of the lockdown.

The MOM training program started on Tuesday 11 February 2020. At the end of the second week of the course, on Sunday 23 February, the regional government of Friuli Venezia-Giulia ordered the closure of all schools and universities. The following six teachings were therefore delivered through 30-minute video lessons sent via the internet, and participants were asked to continue their MOM practice on their own (see Figure 1). They could communicate with their MOM instructor through emails, telephone messages and phone calls in case they wanted to share their meditation experiences or to pose questions about the contents of the video lessons.

Participants were assessed in the days before the training program (Time T0, between 6 February 2020 and 11 February 2020) and after the end of the program (Time T1, between 2 April 2020 and 15 April 2020). Data was obtained using paper-and-pencil questionnaires at T0 and using internet-based questionnaires at T1. For T1, the teachers agreed to complete the survey entirely in one session (as done at T0), paying close attention.

### 2.2. Participants

In total, 67 school teachers (1M, 66F; all Caucasian) were recruited through official communications sent to primary and secondary State schools and preschools in the province of Udine, Friuli Venezia-Giulia. The sample size was determined by the maximum number of participants eligible for the two MOM courses. In the two MOM courses, there was an equal proportion of teachers from different school levels (21 teachers from primary schools, 4 from secondary and 2 from preschools in the first course; 22 from primary, 5 from secondary and 4 from preschools in the second course).

This study concerned the 66 female teachers (age: 51.5 ± 7.9 years) participating in the MOM training program. The sample considered is representative of the Italian teachers, which are on average older than in most other developed countries (average age of teachers in Italy—48.6 years; average for countries of the Organization for Economic Cooperation and Development—44.1 years) [61].

Written informed consent was obtained from all participants during the first assessment session. This study was approved by the Ethics Committee of the University of Udine and all procedures performed in the study were in accordance with the ethical standards of the 1964 Helsinki declaration and its later amendments (ethical code: CGPER-2019-12-09-01). All data was processed anonymously and data confidentiality was ensured.

### 2.3. Mindfulness Oriented Meditation (MOM) Course

The MOM course is an 8-week training program, with a 2 h group meeting per week and 30 min daily meditation practice at home [31,32,36,60,62,63,64]. In its structure it is similar to an MBSR course (Mindfulness-Based Stress Reduction), the most common mindfulness meditation program developed by Kabat-Zinn [65]. Each MOM meeting is divided into three parts: a 15/30 min teaching on topics related to meditative practice, a 30 min guided MOM practice and a final phase during which participants can share their experiences and ask questions to the instructor. These are the topics covered in the teachings [64]: historical introduction to mindfulness meditation, what is mindfulness meditation and how to practice it, facing pain with a mindful attitude, attention and awareness, being in the here and now, disidentification, deautomatization, and letting go. The MOM practice, which is the same throughout the course, entails a 10 min session of paying attention to one’s breathing (anapanasati meditation), 10 min dedicated to heeding one’s bodily sensations (body scan meditation) and 10 min dedicated to being aware of one’s emotional and mental phenomena (vipassana meditation). The main reference for these practices within the Buddhist context, from which mindfulness meditation was adapted, is the Satipatthāna discourse [66], although the instructions given to participants during the meditations do not refer to Buddhism. The same three practices are part of other contemporary, secular and psychologically-oriented mindfulness meditation courses, such as MBSR. After the first meeting, participants are provided with a 30 min audio recording containing a guided MOM practice (http://www.medita-mom.it/materiale), as an aid for home meditation practice. Participants are provided with articles and book suggestions on the topics covered in the course based on personal requests.

The theoretical or practical parts of the MOM course delivered in this study were not tailored for the specific group of teachers participating in the study during the Covid-19 outbreak: the teachings remained adherent to the original version of the MOM course [64] and the meditation—guided by instructors in the first two meetings and to be performed at home by the participants throughout the course—remained the same during the 8 weeks of the course. Discussions between participants and instructors, which normally take place in the final part of each MOM meeting, were replaced by personal communications via mail, phone messages or calls in the last 6 MOM weeks (see Figure 1), after the government prohibited face-to-face meetings due to the Covid-19 outbreak.

### 2.4. Measures

Demographic characteristics were obtained at T0. Participants reported their age, gender, level of education, body height and body weight, previous experiences in meditation and if they were diagnosed with any medical disease or psychiatric disorder.

Home practice was assessed through diaries collected every two weeks. Teachers were asked to record their daily practice of mindfulness meditation in the diaries.

Mindfulness skills were assessed with the Italian version of the Five-Facet Mindfulness Questionnaire (FFMQ [67,68]). This 39-item instrument measures five mindfulness scales: observing (OBS), describing (DES), acting with awareness (AWA), non-judging of inner experience (NJU), and non-reactivity to inner experience (NRE). Respondents are asked to rate each statement using a 5-level Likert scale (1 = never or very rarely true, 5 = very often or always true; item example: “I watch my feelings without getting lost in them”). Higher scores in each scale reflect a greater level of mindfulness. FFMQ has a high internal consistency for the total score (Cronbach’s α = 0.86) and acceptable values for its five scales (α ≥ 0.74) [68] (see below the Results section for present sample Cronbach’s α).

Empathy was assessed with the Italian translation of the Questionnaire of Affective and Cognitive Empathy (QCAE [69,70]). Using a 4-level Likert scale response format (1 = strongly disagree, 4 = strongly agree), this 31-item instrument assesses the two main components of empathy, namely affective empathy (AE, a person’s emotional resonance to other people’s experiences; item example—“It worries me when others are worrying and panicky”) and cognitive empathy (CE, the ability to understand how other people feel; item example—“I always try to consider the other fellow’s feelings before I do something”). QCAE has acceptable internal consistency for the AE and CE components, and the total score (α ≥ 0.77) [70].

The personality profile was assessed with the Italian adaptation of the 125-item Temperament and Character Inventory (TCI; [58,59,71]). This tool assesses the temperament and character dimensions of personality using a dichotomous true-false response format (item example: “I’m often so determined that I continue working long after other people give up”). Temperament scales include novelty-seeking (NS, the propensity towards impulsivity, exploratory excitability and eccentricity), harm avoidance (HA, the propensity to respond strongly to aversive stimuli), reward dependence (RD, the propensity to respond intensely to signs of social gratification) and persistence (P, the tendency to pursue goals despite fatigue and frustration). Character scales include self-directedness (SD, the ability to recognize the self as autonomous, and the feeling of self-confidence, honor and hope), cooperativeness (C, the ability to recognize the self as an organic part of society) and self-transcendence (ST, the ability to recognize the self as an organic part of nature and its source). TCI in its 125-item version has acceptable internal consistency for all scales (α ≥ 0.71) [71].

Interoceptive awareness was assessed with the Italian version of the Multidimensional Assessment of Interoceptive Awareness scale (MAIA [72,73]). This 32-item instrument uses a 6-level Likert scale response format (0 = never, 5 = always; item example—“I notice when I am uncomfortable in my body”). MAIA measures interoceptive awareness through eight subscales: noticing (NOT, the awareness of neutral, comfortable and uncomfortable body sensations), not-distracting (NDI, the propensity not to distract oneself from sensations of discomfort or pain), not-worrying (NWO, the propensity not to worry when sensations of discomfort or pain are experienced), attention regulation (ARE, the ability to focus and sustain attention to body sensations), emotional awareness (EAW, the ability to connect body sensations to underlying emotional states), self-regulation (SRE, the ability to manage distress by listening to body sensations), body listening (BLI, the propensity to actively listen to the body for information) and trusting (TRU, the tendency to trust your body). The Cronbach’s α values for internal consistency varied between 0.53 and 0.80 in the eight MAIA scales [73].

Psychological well-being was assessed with the Italian version of the 18-item Psychological Well-being Scales (PWB [74,75]). This tool uses a 4-level Likert scale response format (1 = strongly disagree, 4 = strongly agree; item example—“I like most parts of my personality”) and the six scales included are self-acceptance (SA), autonomy (AU), environmental mastery (EM), personal growth (PG), positive relations with others (PR) and purpose in life (PL). Higher scores on any scale indicate greater indices of happiness and psychological well-being. PWB Scales showed Cronbach’s α values ranging from 0.60 to 0.70 [76], which appears to be a sufficient level of internal consistency based on the fact that each subscale comprised only three items.

Emotional distress was assessed using the Italian version of the Hospital Anxiety and Depression Scale (HADS [77,78]). This 14-item instrument uses a 4-level Likert scale response format, with levels that differ depending on the question. Although originally developed to measure anxiety (item example: “Worrying thoughts go through my mind”) and depression (item example: “I look forward with enjoyment to things”) in non-psychiatric clinical settings, the HADS has been extensively employed as an effective tool even in non-clinical populations [79]. It provides separate scores for anxiety, depression and global emotional distress. In a review of the literature, HADS showed a high internal consistency across studies for both anxiety (mean Cronbach’s α = 0.83) and depression measures (mean Cronbach’s α = 0.82) [80].

Teacher burnout was assessed with the Italian translation of the Maslach Burnout Inventory Educators Survey (MBI-ES [81,82]). Using a 7-level Likert scale response format (0 = never, 6 = always; item example—“I feel frustrated by my job”), this 22-item instrument measures three components of burnout in relation to teacher–student interaction: feelings of overwhelming emotional exhaustion (EE), depersonalization and detachment from the job (DP), and a lack of personal or professional accomplishment (PA). Burnout is indicated by high scores for EE and DP, and a low score for PA. MBI-ES has acceptable internal consistency for each of its components (α ≥ 0.76) [82].

The evaluation of the MOM course, also in relation to the Covid-19 outbreak, was obtained at T1. This included six general questions prepared by the authors of the study about the MOM course, and four questions related to the Covid-19 emergency (see Table 1). Each question was rated on a 5-point Likert-type scale (for Q1–Q8, 1 = not at all, 5 = very much; for Q9–Q10, 1 = much worse, 5 = much better).

### 2.5. Statistical Analyses

Before all analyses, missing values in participants’ responses were imputed using the mean score of the whole sample for the corresponding item, since the total percentage of missing values was very low (0.10%).

**Figure 2:**
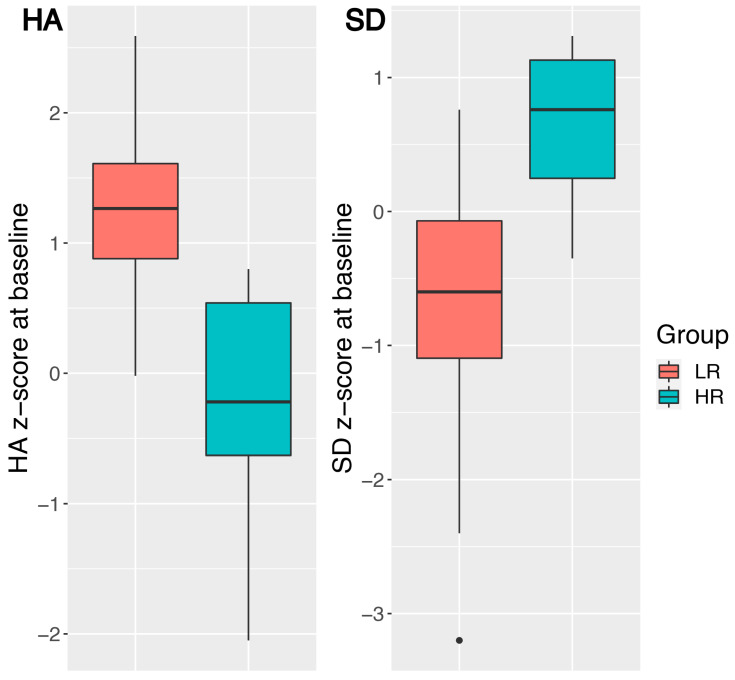
The personality profiles in HA and SD baseline z-scores of the two groups of participants. The LR group (left of each plot, in red) had higher HA and lower SD baseline z-scores than the HR group (right of each plot, in blue). The HA and SD z-scores were obtained from raw scores using a large control sample of healthy individuals (Table S3 in [84]). Abbreviations: HA = Harm Avoidance, LR = Low-Resilience, HR = High-Resilience, SD = Self-Directedness.

Analyses of baseline data and pre- to post-course changes included a between-subject Group factor. As past research has shown that the most influential TCI scales on self-reported resilience were harm avoidance (HA, negatively correlated to resilience) and self-directedness (SD, positively correlated to resilience) [54,55,56], participants were classified as having a low-resilience (LR) or high-resilience (HR) profile on the basis of their HA and SD scores obtained at baseline. Participants in the LR group therefore had higher HA and lower SD scores than participants in the HR group. The clustering procedure, which assigned each participant to one of the two groups, was performed on the participants’ standardized HA and SD scores (see Figure 2) with the k-means algorithm [83].

Between-group differences at baseline were analyzed using independent-sample t tests. Pre- to post-course changes in all the tests were submitted to separate analyses of variance (ANOVAs). All the scales of QCAE, MAIA, PWB, HADS, MBI-ES and the temperament scales of TCI were analyzed, using ANOVAs with Time (Time T0, Time T1) as within-subject and Group (LR, HR) as between-subject factors. While the four temperament scales of TCI are assumed to measure relatively independent aspects of the temperament, the three character scales of TCI are considered to globally measure three aspects of the maturity and development of the self [31], and were therefore analyzed using a single ANOVA with Time (Time T0, Time T1) and Scale (SD, C, ST) as within-subject factors and Group (LR, HR) as the between-subject factor. The same analysis was conducted for FFMQ, where the Scale levels were the five facets of mindfulness (i.e., OBS, DES, AWA, NJU and NRE), as these facets can be considered parts of the unique construct of mindfulness [63].

For these ANOVAs, sensitivity power analyses were carried out as a function of available sample size (with α = 0.05, β = 0.02). These analyses showed that, except for the small main effect of Group in the RD dimension of TCI, the significant effects of interest in this study were greater than the Minimum Detectable Effect’s size (for the main effects of Group, all ηP2 > 0.109, average MDE ηP2 = 0.104; for the main effects of Time, all ηP2 > 0.108, average MDE ηP2 = 0.025; for interaction effects between Time and Group, all ηP2 > 0.081, average MDE ηP2 = 0.014).

The analyses were performed with the free software environment R, version 3.6.3 (R Foundation for Statistical Computing, Vienna, Austria). The assumptions of normality and homogeneity of variance were checked for each group of data (using a significance threshold of 0.01 to avoid alpha-inflation). Data transformation or robust tests were used in the case of violations of the normality assumption. In the case of violations of the sphericity assumption, the degrees of freedom in the ANOVA were corrected by applying the Greenhouse–Geisser procedure (indicated by [GG] in the results). All post-hoc pairwise contrasts were performed using the Holm–Bonferroni procedure. As a measure of effect size, the Partial Eta-Squared (ηP2) was used. All effects are reported as significant at *p* < 0.05.

## 3. Results

Eight teachers could not complete the training, and this resulted in an 11.9% drop-out rate. The final sample consisted of 58 female teachers (age: 50.8 ± 8.0 years).

### 3.1. Objective 1: Baseline Differences in Teachers with LR and HR Personality Profiles

#### Sample Partitioning Based on Resilience Profile

The k-means algorithm included 26 of the 58 participants who completed the MOM course in the high-resilience (HR) profile group and the remaining 32 participants in the low-resilience (LR) profile group (Figure 2). The solution found was stable (i.e., the same classification was invariably obtained by repeating the clustering procedure). When comparing the resilience profiles of the 58 teachers who completed the MOM course with those of the 8 teachers who dropped out (HA: z = −0.20 ± 0.58; SD: z = 0.25 ± 0.40), it was observed that the HA and SD scores of the drop-outs mainly fell in between those of the teachers in the HR group (HA: z = −0.26 ± 0.82; SD: z = 0.64 ± 0.51) and those of the LR group (HA: z = 1.25 ± 0.56; SD: z = −0.77 ± 0.93). The resilience profiles of the 8 teachers who dropped out of the MOM course were not included in the clustering procedure.

The LR and HR groups were significantly different in most of the baseline measurements (see Table 2). First of all, they were different in terms of HA and SD z-scores; this means that the clustering procedure could successfully separate the participants according to their HA and SD features. Moreover, participants in the HR had significantly higher baseline scores in the AWA, NJU and NRE facets of the FFMQ, lower AE scores in the QCAE, higher scores in the NWO and SRE scales of the MAIA, higher scores in all scales of the PWB, lower anxiety and depression scores in the HADS, and lower EE and higher DP and PA scores in the MBI (Hypothesis H1).

### 3.2. Objective 2: Pre–Post MOM Changes in Teachers with LR and HR Personality Profiles

#### 3.2.1. Mindfulness Skills

The raw scores in each FFMQ scale were normalized by dividing the observed scores by the number of items in each scale (eight items for each scale except for the NJU scale, which has seven items). The overall sample total FFMQ score’s Cronbach’s α was 0.91 (time T0) and 0.93 (time T1).

The ANOVA on the normalized FFMQ scores highlighted three significant main effects: a main effect of Time (F(1,56) = 9.7, *p* = 0.003, ηP2 = 0.303) with increased scores at Time T1 (vs. T0), a main effect of Group (F(1,56) = 13.6, p[GG] < 0.001, ηP2 = 0.195) with increased scores in the HR group (vs. LR), and a main effect of Scale (F(4,224) = 5.4, p[GG] = 0.001, ηP2 = 0.203), with significant differences in between-scale scores. A significant interaction effect between Time and Scale was found (F(4,224) = 2.8, p[GG] = 0.03, ηP2 = 0.048), due to different improvements over time in the five scales. Other two- or three-way interactions were not significant (all F < 2.4, *p* > 0.12, ηP2 < 0.041).

#### 3.2.2. Empathy

The overall sample QCAE score’s Cronbach’s α was 0.67 (time T0) and 0.85 (time T1) for AE and 0.76 (time T0) and 0.80 (time T1) for CE.

In the AE scores, the ANOVA revealed a significant main effect of Time (F(1,56) = 17.5, *p* < 0.001, ηP2 = 0.238) with an overall decrease in scores from T0 to T1, and a significant main effect of Group (F(1,56) = 9.9, *p* = 0.003, ηP2 = 0.150), with higher scores in the LR group (vs. HR). There were no other significant main effects or interactions in the AE or in the CE scores (all F(1,56) < 1.6, *p* > 0.21, ηP2 < 0.028).

#### 3.2.3. Personality Profile

Individual raw scores on each TCI scale were converted to z-scores using the age- and gender-matched average scores of a control sample consisting of 320 healthy individuals (Table S3 in [84]).

The overall sample TCI temperament score’s Cronbach’s α was 0.74 (time T0) and 0.68 (time T1) for NS, 0.85 (time T0) and 0.82 (time T1) for HA, 0.65 (time T0) and 0.51 (time T1) for RD, and 0.58 (time T0) and 0.53 (time T1) for P. In the temperament scales of TCI, the ANOVAs revealed a main effect of Group in the HA and RD scales (F(1,56) = 65.7, *p* < 0.001, ηP2 = 0.540 for HA; F(1,56) = 6.0, *p* = 0.02, ηP2 = 0.097 for RD). Participants of the LR group had higher scores than participants in the HR group in both scales. A significant main effect of Time was also observed in the HA scale (F(1,56) = 28.6, *p* < 0.001, ηP2 = 0.338), with an overall decrease in HA scores from T0 to T1.

The overall sample TCI character score’s Cronbach’s α was 0.80 (time T0) and 0.80 (time T1). The ANOVA on the character scales of TCI highlighted three significant main effects: a main effect of Group (F(1,56) = 12.8, *p* < 0.001, ηP2 = 0.186), with higher scores in the HR group (vs. LR), a main effect of Time (F(1,56) = 23.7, *p* < 0.001, ηP2 = 0.298), with an overall increase in scores from T0 to T1, and a main effect of Scale (F(2,112) = 6.8, p[GG] = 0.003, ηP2 = 0.108), with the C and ST scores being greater than the SD scores (z = 4.9, *p* < 0.001 and z = 5.2, *p* < 0.001, respectively). The ANOVA also highlighted two significant interaction effects: an interaction effect between Group and Scale (F(2,112) = 8.2, p[GG] < 0.001, ηP2 = 0.127), with a between-group difference in SD scores but not in C and ST scores, and an interaction effect between Group and Time (F(1,56) = 5.6, *p* = 0.02, ηP2 = 0.090), with scores improving over time more in the LR (χ²(1) = 29.1, *p* < 0.001) than in the HR group (χ²(1) = 2.9, *p* = 0.09).

#### 3.2.4. Interoceptive Awareness

The overall sample MAIA score’s Cronbach’s α was 0.90 (time T0) and 0.90 (time T1). Significant main effects of Group were found in the NWO and SRE scales (F(1,56) = 8.2, *p* = 0.006, ηP2 = 0.128; F(1,56) = 9.0, *p* = 0.004, ηP2 = 0.138, respectively), where scores were higher in the HR than in the LR group.

Significant main effects of Time were found in six of the eight MAIA scales (NOT, ARE, EAW, SRE, BLI, TRU) (for all F(1,56) > 7.6, *p* < 0.008, ηP2 > 0.120), with an increase of scores from T0 to T1. All other main effects and interactions were not significant (for all F(1,56) < 2.7, *p* > 0.11, ηP2 < 0.046).

#### 3.2.5. Psychological Well-Being

The overall sample PWB score’s Cronbach’s α was 0.85 (time T0) and 0.85 (time T1). Significant main effects of Group were found in all PWB scales except PR (for all F(1,56) > 5.8, *p* < 0.02, ηP2 > 0.109): scores in the SA, AU, EM, PG and PL scales were higher in the HR vs. LR group. Significant main effects of Time were observed in the AU, EM and PR scales (for all F(1,56) > 7.9, *p* < 0.007, ηP2 > 0.124), with an increase of scores from T0 to T1. A significant interaction effect between Group and Time was observed in the PR scale (F(1,56) = 8.7, *p* = 0.005, ηP2 = 0.134), with scores improving over time significantly in the LR (χ²(1) = 25.6, *p* < 0.001) but not in the HR group (χ²(1) = 0.3, *p* = 0.55).

#### 3.2.6. Emotional Distress

The overall sample HADS score’s Cronbach’s α was 0.84 (time T0) and 0.81 (time T1). Significant main effects of Group were found in the anxiety and depression scores (F(1,56) = 14.2, *p* < 0.001, ηP2 = 0.202; F(1,56) = 9.1, *p* = 0.004, ηP2 = 0.139, respectively): scores in the LR were higher than in the HR group. Significant main effects of Time were also observed in the anxiety and depression scores (F(1,56) = 8.7, *p* = 0.005, ηP2 = 0.135; F(1,56) = 13.8, *p* < 0.001, ηP2 = 0.198, respectively), with an overall decrease in scores from T0 to T1. A significant interaction effect between Group and Time was observed in depression scores (F(1,56) = 5.3, *p* = 0.03, ηP2 = 0.086), corresponding to a significant decrease in scores in the LR group from T0 to T1 (χ²(1) = 20.1, *p* < 0.001), but not in the HR group. Due to the different decrease of depression scores in the two groups, the between-group difference in depression scores was significant at T0 (χ²(1) = 13.6, *p* < 0.001), but not at T1 (χ²(1) = 3.2, *p* = 0.07).

#### 3.2.7. Teacher Burnout

The overall sample MBI score’s Cronbach’s α was 0.88 (time T0) and 0.87 (time T1). Significant main effects of Group were found in the EE component of burnout (F(1,56) = 17.7, *p* < 0.001, ηP2 = 0.240), with higher scores in the LR (vs. HR) group, and in the PA component of burnout (F(1,56) = 10.4, *p* = 0.002, ηP2 = 0.157), with higher scores in the HR (vs. LR) group. A significant main effect of Time was observed in the EE component (F(1,56) = 6.7, *p* = 0.01, ηP2 = 0.108), with an overall decrease in scores from T0 to T1. All other main effects and interactions were not significant (for all F(1,56) < 2.6, *p* > 0.11, ηP2 < 0.044).

#### 3.2.8. Adherence to Practice

Each teacher reportedly meditated on average for 1307.7 ± 22.5 min during the MOM course (i.e., about 26 min per day). The teachers in the HR group reportedly meditated more than teachers in the LR group, but this difference was not significant (average meditation practice during the MOM course in the LR group: 1277.4 ± 220.9 min; in the HR group: 1338.4 ± 224.2 min).

In sum, the data on the pre–post MOM change showed that both groups significantly improved in terms of mindfulness skills, affective empathy, harm avoidance, character traits (with greater improvements in the low-resilience group) and interoceptive awareness, supporting Hypothesis H2. Both groups significantly improved also in terms of psychological well-being (in the scales of autonomy, environmental mastery and positive relations with others), anxiety, depression (with greater improvements in the low-resilience group), and in the emotional exhaustion scale of burnout.

### 3.3. Objective 3: Teachers’ Satisfaction with the MOM Course (in General and in the Context of the Covid-19 Outbreak)

#### Evaluation of the MOM Course

When asked at T1 about their satisfaction with the course (Q1–Q6, see Table 1), the participants reported that they generally enjoyed the course (average score: 4.1, SD = 0.7), that they found it rather challenging (M = 3.7, SD = 0.9) but quite useful (M = 4.0, SD = 0.7), that they considerably engaged in it (M = 3.9, SD = 0.6), that the course moderately met their professional training needs (M = 3.6, SD = 0.8) and that the instructors’ competence was very good (M = 4.5, SD = 0.6). A significant between-group difference was found only in the question about the usefulness of the course (in the LR group: M = 3.81, SD = 0.69; in the HR group: M = 4.15, SD = 0.73; Fisher’s exact test *p* = 0.046).

In the questions about the course in the context of the Covid-19 lockdown (Q7–Q10, see Table 1), the participants reported that thinking about Covid-19 distracted them slightly during practice sessions (M = 2.5, SD = 0.9), that the practice of meditation helped them very much during the particular period of isolation related to the emergency (M = 3.6, SD = 0.7), that the absence of the practice would have made the period a little worse (M = 1.9, SD = 0.6) and that the distance learning methods of the last six meetings of the MOM course somewhat reduced the effectiveness of the course (M = 2.1, SD = 0.7). No significant between-group differences were found in these questions (for all, Fisher’s exact test *p* > 0.26).

Finally, we investigated whether there was any between-group difference in the relationship between the responses given in the questions concerning the MOM course in the context of the Covid-19 outbreak (Q7–Q10) and the pre/post changes in some of the tests used (namely TCI, PWB and HADS, the tests where a significant interaction between Group and Time was detected in the ANOVAs). This analysis was performed using multinomial logistic regressions, separately for each question, using Group membership (LR, HR) and responses (properly dichotomized into two levels) to the question as predictor variables, and changes in the test scores from before to after the MOM course as outcome variables. A significant result was found only for question Q9 (“How do you think you would have lived this period without the practice?”), the responses to which were dichotomized with ‘0’ for “much worse” responses and ‘1’ for responses from “somewhat worse” to “much better”, in relationship with the HADS Anxiety change scores. The pre/post MOM change in the Anxiety scores was predicted, by Q9 responses, differently in the two groups (b = −0.81, *p* = 0.02): in the LR group the changes in the Anxiety scores were not related to Q9 responses (r = 0.10, *p* = 0.57), while in the HR group these changes were negatively related to Q9 responses (r = −0.53, *p* = 0.005). This means that, for the HR group only, the more the Anxiety scores decreased, the more participants felt that they would have had more difficulty living through the lockdown period without the meditation practice.

## 4. Discussion

In the present study, a sample of female teachers was assessed one month before and one month after the imposition of the Covid-19 lockdown in Italy, and received a mindfulness meditation intervention during these two months. The personality profiles of the participants at baseline were used to classify the participants into a low-resilience group and a high-resilience group. The scores obtained from the teachers in the self-report measures were studied as a function of time (pre- vs. post-course) and group (low- vs. high-resilience).

### 4.1. Objective 1: Baseline Differences in Teachers with LR and HR Personality Profiles

Results largely supported H1: the analysis of data at baseline revealed that the low-resilience group had significantly worse average scores than the high-resilience group in terms of personality profiles (by the definition of the two groups), mindfulness skills, affective empathy, interoceptive awareness, psychological well-being, anxiety, depression and burnout levels. This between-group difference is in line with previous research that found resilience to be directly related, on the one hand, to mindfulness [50], psychological well-being [48] and interoceptive awareness [51], and on the other hand, inversely related to burnout [53], anxiety and depression [52].

### 4.2. Objective 2: Pre–Post MOM Changes in Teachers with LR and HR Personality Profiles

The results also supported H2; at the end of the course, one month after the lockdown was imposed, significant improvements were observed in both groups in most of the self-report measures. Both groups had significantly improved scores in mindfulness skills, affective empathy, harm avoidance, character traits (especially participants in the low-resilience group) and interoceptive awareness (H2), as well as in psychological well-being (in the scales of autonomy, environmental mastery and positive relations with others), anxiety, depression (especially in the low-resilience group) and emotional exhaustion. These positive results in the measures used in our study could be related to participants’ adherence to practice (participants’ home practice in our study was about 88% of the assigned amount), which was similar in the two groups and could be classified as good compared to other standard introductory MBIs [85,86]; various studies have indeed highlighted a direct dose–response relationship between the amount of meditation practice and the extent of the beneficial outcomes of MBIs (for a meta-analysis, see [87]), and in the case of online MBIs as well (for a meta-analysis, see [37]). It is indeed worth pointing out that mindfulness meditation was taught here with two face-to-face initial meeting and video lessons sent via internet for the next six weeks of the course (see Figure 1); our results therefore confirm the possibility of effectively delivering MBIs via the internet [37,38].

During the early stages of the Covid-19 epidemic, many researchers reported the need to provide interventions to support the mental health of people affected by the Covid-19 crisis [15,16,19,87,88], in particular for healthcare workers [18,89,90,91] and patients with the Covid-19 infection [92,93]. For this purpose, many scholars suggested the use of mindfulness interventions [94,95,96,97]. However, studies assessing the impact of interventions that supported mental health in the early stages of the Covid-19 related crisis are very limited. Although the improvements reported in the present study cannot be attributed to the MOM course given the absence of a control group, the results seem remarkable, as we are not aware of any studies reporting improved psychological well-being when comparing persons during the Covid-19 outbreak with their precedent condition. Various studies instead reported a deterioration of people’s mental health [5,6,98,99]. At the same time, it should be recognized that our research focused on a specific population, namely state school teachers, who might have been less impacted by the emergency than other workers, as their jobs were not at risk and they were not professionally exposed to the risk of the infection (as were, for example, health care workers). Although a recent meta-analysis on the psychological impact of Covid-19 on medical staff and the general public found that the prevalence of anxiety and depression was similar in these two groups [100], there is evidence that certain occupations, such as front-line nurses, were indeed more psychologically affected by Covid-19 than others [101,102].

In our study, for most of the measurements where the improvements were observed, the rate of change over time was generally similar in the two groups of teachers differing in terms of resilience. Since the two groups were different at baseline in most of the measurements, the final data generally showed that the low-resilience and the high-resilience groups were still different after the MOM course in the dimensions in which they differed at baseline (mindfulness skills, affective empathy, harm avoidance, the self-regulation scale of interoceptive awareness, the autonomy and environmental mastery scales of psychological well-being, anxiety and emotional exhaustion) and still similar in the dimensions for which they were comparable at baseline (the noticing, attention regulation, emotional awareness, body listening and trusting scales of interoceptive awareness). This pattern was different in the “positive relation with others” (PR) scale of psychological well-being, in character traits, in depression scores and in the total score of emotional distress, wherein the low-resilience individuals showed greater improvement than the high-resilience individuals (see the Time X Group interactions reported in Table 3). LR individuals therefore made better progresses than HR participants, although both groups reported the same amount of home meditation practice. This could be due, at least in part, to ceiling effects, because highly resilient individuals already showed healthy profiles at baseline for these measures. The findings could also be linked to different levels of motivation, across HR and LR participants, to commit themselves to the mindfulness intervention, which is usually presented as a way to alleviate one’s psychological suffering [103]; as described in our previous research [36] and similarly to the reactive control negative feedback process proposed in many theories of self-regulation [104], participants who are in a worse condition (i.e., the LR group in our study) could be more motivated to engage for their own recovery.

It is important to underline that the scores for the personality traits used to define resilience in our study (i.e., harm avoidance and self-directedness) also improved in both LR and HR groups in the present study. Resilience is indeed considered a modifiable characteristic [105] and, during the Covid-19 pandemic, it has been related to modifiable factors such as physical exercise and interpersonal relationships [106]. However, as observed in other measures of the present research, at the end of the training course the two groups in our study remained significantly different in terms of the two aforementioned personality traits, and therefore in terms of resilience.

### 4.3. Objective 3: Teachers’ Satisfaction with the MOM Course (in General and in the Context of the Covid-19 Outbreak)

The answers to the questions about general satisfaction with the MOM course supported H3a; teachers generally enjoyed the course, found it challenging but useful, engaged in it and appreciated the work of the instructors and the course content for their professional training needs. In addition to these positive responses from the teachers who completed the course, the teachers’ satisfaction with the course itself can also be expressed by the number of teachers who dropped out (11.9% of drop-out in our study), which, in the present study, can be classified as medium-to-low when compared to the corresponding past literature [30,107,108,109]. The answers to the questions about the course in the context of the Covid-19 outbreak supported H3b; participants in general reported that the practice of meditation helped them during the particular period of isolation related to the health emergency, and that the absence of the practice would have made the period a little worse.

Finally, teachers’ evaluation of the MOM course was analyzed in relation to the two different resilience profiles of participants. Some between-group differences were observed. Although teachers generally appreciated the course and actively engaged in it, those in the low-resilience group reported significantly lower scores than their colleagues in the high-resilience group when asked about the usefulness of the course. This is quite surprising, because both groups equally benefited from the course, and the low-resilience group even had greater improvements in the emotional distress and character traits scores. Moreover, for the question on how one would have lived during the Covid-19 lockdown period without practicing mindfulness meditation, only teachers in the high-resilience group gave answers that appeared to be coherent with their self-reported decrease in anxiety levels: the more the anxiety scores decreased after vs. before the MOM course, the more the participants reported that the same period would have been much worse without the meditation practice. Both aspects that emerged from the answers to these questions suggest that less resilient individuals could have been less able to explicitly recognize the positive changes following the MOM training during the emergency situation. This should be taken into account in future mindfulness interventions in this type of context, in particular to motivate people to continue to use the learned technique even after the end of the intervention.

### 4.4. Limitations

Some limitations should be noted in our study. First, both LR and HR groups received the same intervention; for the generalizability of our findings, a control group should have been employed. Second, again in relation to the issue of generalizability, future studies should include samples with younger participants than those included in our study, because younger teachers may react to states of emergency, such as the Covid-19 pandemic, differently than older teachers. Third, only self-report measures were used, which can be susceptible to socially desirable responding. Moreover, the questions about the evaluation of the MOM course were not taken from a validated instrument: in order to further explore how participants find and accept psychological/behavioral interventions, future studies could benefit from using instruments such as the theoretical framework of acceptability [110]. A final issue concerns individuals’ spirituality. In different contexts and with different participants, past studies showed, on the one hand, that MOM practice may contribute to enhancing individuals’ levels of implicit and explicit spirituality, [62] and, on the other hand, that spirituality is an important mediator of the positive health effects of mindfulness meditation [111]. Considering the strong connections between spirituality and healthcare (e.g., [112]), it might have been useful to explicitly test spirituality in our study (beyond the self-transcendence scale included in the TCI measuring the predisposition of human beings toward spiritual feeling, thinking and behaviors), and it might be important that future studies investigating the psychological impact of health emergencies, and the effectiveness of related interventions, include the dimension of people’s spirituality (e.g., [113]).

## 5. Conclusions

We have shown that it is possible through mindfulness meditation to enhance the resilience and improve the well-being of school teacher participants during critical events, such as the Covid-19 lockdown in Italy. This was seen to occur both in female individuals with resilient personality profiles and in female individuals with weaker personality traits. The factors that, in our case, may have determined this success were as follows: (1) a consolidated and evidence-based program that was provided by institutional services, (2) the intervention started before the beginning of the pandemic (i.e., in a preventive approach) [114] and (3) the participants actively engaged daily in the practice of meditation to protect and promote their mental health.

## Figures and Tables

**Table 1:** The questions for the teachers’ evaluation of the MOM.

Question	Course Evaluation
Q1	How much did you enjoy the course?
Q2	How challenging was it?
Q3	How useful was it for you?
Q4	How much did you engage in it?
Q5	In relation to training needs and your professional role, to what extent has it met your needs?
Q6	How adequate do you think the instructor’s competence is with regards to content and teaching methods?
	**Context of the course (COVID-19 lockdown)**
Q7	How often have you been distracted by thinking about Covid-19 during practice sessions?
Q8	How much do you think the practice of meditation helped you during this particular period of isolation related to the emergency?
Q9	How do you think you would have lived through this period without the practice?
Q10	How do you think the distance learning methods of the last 6 meetings have conditioned the effectiveness of the course?

**Table 2:** Baseline data (expressed as average score and standard deviation) and between-group statistical differences. For baseline data, raw scores are used for all measures except TCI, for which z-scores are reported (obtained using a large control sample of healthy individuals—see Table S3 in [84]). Text in italics indicates that results were obtained using a robust version of the t test.

Test	Scale	Baseline Score		Difference between Groups	
		LR Group	HR Group	t	*p*
FFMQ	OBS	26.47 (4.81)	28.15 (4.31)	−1.41	0.17
	DES	25.53 (5.00)	27.58 (6.45)	−1.33	0.19
	AWA	23.16 (5.16)	28.08 (5.99)	−3.31	<0.01 *
	NJU	24.19 (5.23)	29.12 (5.71)	−3.39	<0.01 *
	NRE	18.25 (3.72)	22.00 (3.78)	−3.78	<0.01 *
QCAE	CE	57.81 (5.44)	58.54 (6.75)	−0.44	0.66
	AE	39.06 (3.56)	34.62 (4.61)	4.03	<0.01 *
TCI	NS	−0.21 (1.18)	0.24 (1.04)	−1.52	0.13
	HA	1.25 (0.56)	−0.26 (0.82)	7.97	<0.01 *
	RD	0.18 (0.97)	−0.25 (1.06)	1.57	0.12
	P	−0.08 (1.00)	−0.09 (0.91)	*−0.44*	*0.66*
	SD	−0.77 (0.93)	0.64 (0.51)	−7.31	<0.01 *
	C	0.30 (0.65)	0.38 (0.91)	*−1.22*	*0.23*
	ST	0.33 (0.83)	0.64 (1.09)	−1.19	0.24
MAIA	NOT	3.30 (0.93)	3.55 (0.88)	−1.02	0.31
	NDI	2.12 (0.68)	2.13 (0.81)	−0.02	0.99
	NWO	2.08 (0.95)	2.81 (1.06)	−2.71	0.01 *
	ARE	2.15 (0.59)	2.50 (0.85)	−1.79	0.08
	EAW	3.60 (0.94)	3.62 (0.85)	−0.10	0.92
	SRE	2.38 (0.98)	3.03 (0.70)	−2.93	<0.01 *
	BLI	2.30 (1.03)	2.51 (0.91)	−0.82	0.41
	TRU	2.82 (1.20)	3.23 (1.25)	−1.26	0.21
PWB	SA	11.38 (3.10)	13.88 (2.12)	−3.65	<0.01 *
	AU	9.69 (2.93)	12.73 (2.74)	−4.08	<0.01 *
	EM	9.91 (2.68)	12.08 (3.16)	−2.78	0.01 *
	PG	14.94 (1.98)	16.23 (1.82)	*−3.02*	0.01 *
	PR	12.06 (2.69)	14.12 (3.12)	−2.65	0.01 *
	PL	12.09 (3.00)	14.73 (2.71)	−3.52	<0.01 *
HADS	Anxiety	9.69 (3.41)	6.12 (3.98)	3.62	<0.01 *
	Depression	5.19 (2.47)	3.00 (1.88)	3.83	<0.01 *
	Total Score	14.88 (5.25)	9.12 (4.89)	4.31	<0.01 *
MBI	EE	25.69 (11.63)	16.04 (9.44)	3.49	<0.01 *
	DP	3.53 (3.75)	1.81 (2.65)	*2.12*	0.04 *
	PA	35.78 (5.51)	40.62 (5.40)	−3.36	<0.01 *

Asterisks (*) indicate significant difference (*p* < 0.05). Values in italics were obtained by using data transformation or robust tests (see Section 2.5). Abbreviations: LR = Low-Resilience, HR = High-Resilience, FFMQ = Five-Facet Mindfulness Questionnaire (OBS = Observe, DES = Describe, AWA = Act with awareness, NJU = Non judge, NRE = Non react), QCAE = Questionnaire of Cognitive and Affective Empathy (AE = Affective Empathy, CE = Cognitive Empathy), TCI = Temperament and Character Inventory (NS = Novelty Seeking, HA = Harm Avoidance, RD = Reward Dependence, P = Persistence, SD = Self-Directedness, C = Cooperativeness, ST = Self-Transcendence), MAIA = Multidimensional Assessment of Interoceptive Awareness (NOT = Noticing, NDI = Not-Distracting, NWO = Not-Worrying, ARE = Attention Regulation, EAW = Emotional Awareness, SRE = Self-Regulation, BLI = Body Listening), PWB = Psychological Well-being (SA = Self-Acceptance, AU = Autonomy, EM = Environmental Mastery, PG = Personal Growth, PR = Positive Relations with others, PL = Purpose in Life), HADS = Hospital Anxiety and Depression Scale, MBI = Maslach Burnout Inventory (EE = Emotional Exhaustion, DP = Depersonalization, PA = Personal Accomplishment).

**Table 3:** Pre/post change in the scores (calculated as post score minus pre score; expressed in the table as average change and standard deviation) and results of the ANOVAs (detailed for the main effect of Group, the main effect of Time and the interaction effect between Group and Time). For TCI, z-scores are used (obtained as in Table 2). Text in italics indicates that results were obtained using a robust version of ANOVA. Abbreviations are as in Table 2.

Test	Scale	Pre-Post Change		2-Way Mixed Model ANOVA					
		LR Group	HR Group	Group		Time		Group: Time	
				F	*p*	F	*p*	F	*p*
FFMQ	OBS	3.78 (4.20)	2.69 (3.03)	1.2	0.28	43.3	<0.01 *	1.2	0.27
	DES	1.84 (5.16)	0.69 (3.07)	0.9	0.33	4.9	0.03 *	1.0	0.32
	AWA	3.06 (4.70)	1.08 (3.88)	8.4	0.01 *	13.0	<0.01 *	3.0	0.09
	NJU	2.38 (4.98)	1.54 (3.73)	13.0	<0.01 *	11.0	<0.01 *	0.5	0.48
	NRE	1.72 (4.71)	0.73 (3.79)	21.6	<0.01 *	4.6	0.04 *	0.7	0.39
QCAE	CE	−0.97 (5.20)	−0.62 (4.03)	0.4	0.54	1.6	0.21	0.1	0.78
	AE	−2.97 (4.40)	−1.65 (3.92)	9.9	<0.01 *	17.5	<0.01 *	1.4	0.24
TCI	NS	0.05 (0.77)	−0.19 (0.57)	1.4	0.23	0.6	0.43	1.8	0.19
	HA	−0.46 (0.33)	−0.23 (0.64)	65.7	<0.01 *	28.6	<0.01 *	3.1	0.08
	RD	0.04 (0.64)	−0.22 (0.68)	6.0	0.02 *	1.1	0.29	2.3	0.14
	P	0.19 (0.95)	0.05 (0.74)	*<0.1*	*0.89*	*0.8*	*0.39*	*0.5*	*0.50*
	SD	0.64 (0.74)	0.12 (0.49)	39.0	<0.01 *	20.1	<0.01 *	9.3	<0.01 *
	C	0.24 (0.63)	0.17 (0.64)	*0.7*	*0.42*	*4.4*	0.04 *	*2.3*	*0.14*
	ST	0.36 (0.66)	0.13 (0.47)	0.6	0.44	10.2	<0.01 *	2.2	0.15
MAIA	NOT	0.52 (0.77)	0.42 (0.54)	1.2	0.28	27.4	<0.01 *	0.3	0.61
	NDI	−0.08 (0.75)	−0.28 (1.00)	0.6	0.44	2.5	0.12	0.8	0.39
	NWO	0.18 (1.07)	−0.22 (0.86)	8.2	0.01 *	<0.1	0.87	2.3	0.13
	ARE	1.03 (0.71)	0.76 (0.92)	2.7	0.11	69.9	<0.01 *	1.6	0.21
	EAW	0.33 (0.78)	0.27 (0.88)	<0.1	0.96	7.6	0.01 *	0.1	0.78
	SRE	0.96 (0.94)	0.62 (0.81)	9.0	<0.01 *	46.4	<0.01 *	2.1	0.15
	BLI	0.86 (1.08)	0.67 (0.89)	0.3	0.56	33.5	<0.01 *	0.5	0.46
	TRU	0.74 (0.88)	0.44 (0.88)	1.0	0.32	25.5	<0.01 *	1.7	0.20
PWB	SA	0.59 (2.85)	−0.35 (2.06)	*4.4*	0.4 *	*0.2*	*0.62*	*3.0*	*0.09*
	AU	0.97 (3.04)	1.00 (2.06)	20.2	<0.01 *	7.9	0.01 *	<0.1	0.96
	EM	1.50 (2.26)	1.04 (2.78)	9.0	<0.01 *	14.7	<0.01 *	0.5	0.49
	PG	−0.03 (3.04)	−0.08 (1.74)	*5.8*	0.02 *	*0.1*	*0.73*	*0.9*	*0.34*
	PR	1.78 (1.88)	0.23 (2.12)	3.1	0.08	14.6	<0.01 *	8.7	<0.01 *
	PL	0.72 (1.85)	−0.38 (2.56)	9.0	<0.01 *	0.3	0.57	3.6	0.06
HADS	Anxiety	−1.53 (2.68)	−0.54 (2.63)	14.2	<0.01 *	8.7	<0.01 *	2.0	0.16
	Depression	−1.47 (2.09)	−0.35 (1.50)	9.1	<0.01 *	13.8	<0.01 *	5.3	0.03 *
	Total Score	−3.00 (4.13)	−0.88 (2.82)	16.0	<0.01 *	16.7	<0.01 *	4.9	0.03 *
MBI	EE	−2.56 (9.47)	−2.92 (5.67)	17.7	<0.01 *	6.7	0.01 *	<0.1	0.87
	DP	−0.62 (4.09)	1.00 (4.08)	*2.5*	*0.12*	*0.3*	*0.56*	*1.0*	*0.32*
	PA	−0.94 (6.44)	−1.42 (4.23)	10.4	<0.01 *	2.6	0.11	0.1	0.74

Asterisks (*) indicate significant difference (*p* < 0.05). Values in italics were obtained by using data transformation or robust tests (see Section 2.5).

## References

[1] World Health Organization (2020). Coronavirus Disease 2019 (COVID-19).

[2] World Health Organization (2020). Coronavirus Disease 2019 (COVID-19).

[3] International Monetary Fund (2020). Italy: 2020 Article IV Consultation-Press Release; Staff Report; and Statement by the Executive Director for Italy.

[4] Moccia L., Janiri D., Pepe M., Dattoli L., Molinaro M., De Martin V., Di Nicola M. (2020). Affective temperament, attachment style, and the psychological impact of the COVID-19 outbreak: An early report on the Italian general population. Brain Behav. Immun..

[5] Favieri F., Forte G., Tambelli R., Casagrande M. (2020). The Italians in the time of coronavirus: Psychosocial aspects of unexpected COVID-19 pandemic. Lancet.

[6] Davico C., Ghiggia A., Marcotulli D., Ricci F., Amianto F., Vitiello B. (2020). Psychological impact of the COVID-19 pandemic on adults and their children in Italy. Lancet.

[7] Huang Y., Zhao N. (2020). Generalized anxiety disorder, depressive symptoms and sleep quality during COVID-19 epidemic in China: A web-based cross-sectional survey. Psychiatry Res..

[8] Wang C., Pan R., Wan X., Tan Y., Xu L., Ho C.S., Ho R.C. (2020). Immediate psychological responses and associated factors during the initial stage of the 2019 Coronavirus disease (COVID-19) epidemic among the general population in China. Int. J. Environ. Res. Public Health.

[9] Cao W., Fang Z., Hou G., Han M., Xu X., Dong J., Zheng J. (2020). The psychological impact of the COVID-19 epidemic on college students in China. Psychiatry Res..

[10] Sun L., Sun Z., Wu L., Zhu Z., Zhang F., Shang Z., Liu W. (2020). Prevalence and risk factors of acute posttraumatic stress symptoms during the COVID-19 outbreak in Wuhan, China. MedRxiv.

[11] Brooks S.K., Webster R.K., Smith L.E., Woodland L., Wessely S., Greenberg N., Rubin G.J. (2020). The psychological impact of quarantine and how to reduce it: Rapid review of the evidence. Lancet.

[12] Gentilini U., Almenfi M., Orton I., Dale P. (2020). Social Protection and Jobs Responses to Covid-19: A Real-Time Review of Country Measures.

[13] Logie C.H., Turan J.M. (2020). How do we balance tensions between COVID-19 public health responses and stigma mitigation? Learning from HIV research. AIDS Behav..

[14] Geoffroy P.A., Le Goanvic V., Sabbagh O., Richoux C., Weinstein A., Dufayet G., Lejoyeux M. (2020). Psychological support system for hospital workers during the COVID-19 outbreak: Rapid design and implementation of the Covid-psy hotline. Front. Psychiatry.

[15] Zhou X. (2020). Psychological crisis interventions in Sichuan Province during the 2019 novel coronavirus outbreak. Psychiatry Res..

[16] Pfefferbaum B., North C.S. (2020). Mental health and the COVID-19 pandemic. N. Engl. J. Med..

[17] Liu S., Yang L., Zhang C., Xiang Y.T., Liu Z., Hu S., Zhang B. (2020). Online mental health services in China during the COVID-19 outbreak. Lancet Psychiatry.

[18] Yang L., Yin J., Wang D., Rahman A., Li X. (2020). Urgent need to develop evidence-based self-help interventions for mental health of healthcare workers in COVID-19 pandemic. Psychol. Med..

[19] Zhou X., Snoswell C.L., Harding L.E., Bambling M., Edirippulige S., Bai X., Smith A.C. (2020). The role of telehealth in reducing the mental health burden from COVID-19. Telemed. E Health.

[20] Fischer R., Karl J.A., Bortolini T., Zilberberg M., Robinson K., Rabelo A.L.A., Mattos P. (2020). Rapid review and meta-meta-analysis of self-guided interventions to address anxiety, depression and stress during COVID-19 social distancing. PsyArXiv.

[21] Chiesa A., Malinowski P. (2011). Mindfulness-based approaches: Are they all the same?. J. Clin. Psychol..

[22] Kabat-Zinn J. (2003). Mindfulness-based interventions in context: Past, present, and future. Clin. Psychol..

[23] Chiesa A., Serretti A. (2006). Mindfulness-based stress reduction for stress management in healthy people: A review and meta-analysis. J. Altern. Complement. Med..

[24] Hofmann S.G., Sawyer A.T., Witt A.A., Oh D. (2010). The effect of mindfulness-based therapy on anxiety and depression: A meta-analytic review. J. Consult. Clin. Psychol..

[25] Goldberg S., Tucker R.P., Greene P.A., Davidson R.J., Wampold B.E., Kearney D.J., Simpson T.L. (2018). Mindfulness-based interventions for psychiatric disorders: A systematic review and meta-analysis. Clin. Psychol. Rev..

[26] Luken M., Sammons A. (2016). Systematic review of mindfulness practice for reducing job burnout. Am. J. Occup. Ther..

[27] Gawande R., To M.N., Pine E., Griswold T., Creedon T.B., Brunel A., Schuman-Olivier Z. (2019). Mindfulness training enhances self-regulation and facilitates health behavior change for primary care patients: A randomized controlled trial. J. Gen. Intern. Med..

[28] Matiz A., Guzzon D., Crescentini C., Paschetto A., Fabbro F. (2020). The role of self body brushing vs mindfulness meditation on interoceptive awareness: A non-randomized pilot study on healthy participants with possible implications for body image disturbances. Eur. J. Integr. Med..

[29] Birnie K., Speca M., Carlson L.E. (2010). Exploring self-compassion and empathy in the context of mindfulness-based stress reduction (MBSR). Stress Health.

[30] Lamothe M., Rondeau E., Malboeuf-Hurtubise C., Duval M., Sultan S. (2016). Outcomes of MBSR or MBSR-based interventions in health care providers: A systematic review with a focus on empathy and emotional competencies. Complement. Ther. Med..

[31] Campanella F., Crescentini C., Urgesi C., Fabbro F. (2014). Mindfulness-oriented meditation improves self-related character scales in healthy individuals. Compr. Psychiatry.

[32] Crescentini C., Matiz A., Fabbro F. (2015). Improving personality/character traits in individuals with alcohol dependence: The influence of mindfulness-oriented meditation. J. Addict. Dis..

[33] Brown K.W., Ryan R.M. (2003). The benefits of being present: Mindfulness and its role in psychological well-being. J. Pers. Soc. Psychol..

[34] Carmody J., Baer R.A. (2008). Relationships between mindfulness practice and levels of mindfulness, medical and psychological symptoms and well-being in a mindfulness-based stress reduction program. J. Behav. Med..

[35] Mantzios M., Giannou K. (2014). Group vs. single mindfulness meditation: Exploring avoidance, impulsivity, and weight management in two separate mindfulness meditation settings. Appl. Psychol. Health Well Being.

[36] Matiz A., Fabbro F., Crescentini C. (2017). Single vs. group mindfulness meditation: Effects on personality, religiousness/spirituality, and mindfulness skills. Mindfulness.

[37] Spijkerman M.P.J., Pots W.T.M., Bohlmeijer E.T. (2016). Effectiveness of online mindfulness-based interventions in improving mental health: A review and meta-analysis of randomised controlled trials. Clin. Psychol. Rev..

[38] Sevilla-Llewellyn-Jones J., Santesteban-Echarri O., Pryor I., McGorry P., Alvarez-Jimenez M. (2018). Web-based mindfulness interventions for mental health treatment: Systematic review and meta-analysis. JMIR Ment. Health.

[39] Casagrande M., Favieri F., Tambelli R., Forte G. (2020). The enemy who sealed the world: Effects quarantine due to the COVID-19 on sleep quality, anxiety, and psychological distress in the Italian population. Sleep Med..

[40] Broche-Pérez Y., Fernández-Fleites Z., Jiménez-Puig E., Fernández-Castillo E., Rodríguez-Martin B.C. (2020). Gender and fear of COVID-19 in a Cuban population sample. Int. J. Ment. Health Addict..

[41] Mazza C., Ricci E., Biondi S., Colasanti M., Ferracuti S., Napoli C., Roma P. (2020). A Nationwide survey of psychological distress among Italian people during the COVID-19 pandemic: Immediate psychological responses and associated factors. Int. J. Environ. Res. Public Health.

[42] Liu N., Zhang F., Wei C., Jia Y., Shang Z., Sun L., Liu W. (2020). Prevalence and predictors of PTSS during COVID-19 outbreak in China hardest-hit areas: Gender differences matter. Psychiatry Res..

[43] Southwick S.M., Charney D.S. (2018). Resilience: The Science of Mastering Life’s Greatest Challenges.

[44] Karreman A., Vingerhoets A.J. (2012). Attachment and well-being: The mediating role of emotion regulation and resilience. Personal. Individ. Differ..

[45] Simeon D., Yehuda R., Cunill R., Knutelska M., Putnam F.W., Smith L.M. (2007). Factors associated with resilience in healthy adults. Psychoneuroendocrinology.

[46] Davydov D.M., Stewart R., Ritchie K., Chaudieu I. (2010). Resilience and mental health. Clin. Psychol. Rev..

[47] Hu T., Zhang D., Wang J. (2015). A meta-analysis of the trait resilience and mental health. Personal. Individ. Differ..

[48] Sagone E., De Caroli M.E. (2014). A correlational study on dispositional resilience, psychological well-being, and coping strategies in university students. Am. J. Educ. Res..

[49] Morice-Ramat A., Goronflot L., Guihard G. (2018). Are alexithymia and empathy predicting factors of the resilience of medical residents in France?. Int. J. Med. Educ..

[50] Keye M.D., Pidgeon A.M. (2013). Investigation of the relationship between resilience, mindfulness, and academic self-efficacy. Open J. Soc. Sci..

[51] Haase L., Stewart J.L., Youssef B., May A.C., Isakovic S., Simmons A.N., Paulus M.P. (2016). When the brain does not adequately feel the body: Links between low resilience and interoception. Biol. Psychol..

[52] Hjemdal O., Vogel P.A., Solem S., Hagen K., Stiles T.C. (2010). The relationship between resilience and levels of anxiety, depression, and obsessive-compulsive symptoms in adolescents. Clin. Psychol. Psychother..

[53] Cooke G.P., Doust J.A., Steele M.C. (2013). A survey of resilience, burnout, and tolerance of uncertainty in Australian general practice registrars. BMC Med. Educ..

[54] Kim J.W., Lee H.K., Lee K. (2013). Influence of temperament and character on resilience. Compr. Psychiatry.

[55] Eley D.S., Cloninger C.R., Walters L., Laurence C., Synnott R., Wilkinson D. (2013). The relationship between resilience and personality traits in doctors: Implications for enhancing well being. PeerJ.

[56] Eley D.S., Leung J., Hong B.A., Cloninger K.M., Cloninger C.R. (2016). Identifying the dominant personality profiles in medical students: Implications for their well-being and resilience. PLoS ONE.

[57] Oshio A., Taku K., Hirano M., Saeed G. (2018). Resilience and big five personality traits: A meta-analysis. Personal. Individ. Differ..

[58] Cloninger C.R., Przybeck T.R., Svrakic D.M., Wetzel R.D. (1994). The Temperament and Character Inventory (TCI): A guide to Its Development and Use.

[59] Cloninger C.R., Svrakic D.M., Przybeck T.R. (1993). A psychobiological model of temperament and character. Arch. Gen. Psychiatry.

[60] Fabbro A., Fabbro F., Capurso V., D’Antoni F., Crescentini C. (2020). Effects of mindfulness training on school teachers’ self-reported personality traits as well as stress and burnout levels. Percept. Mot. Skills.

[61] OECD (2020). TALIS 2018 Results (Volume II): Teachers and School Leaders as Valued Professionals.

[62] Crescentini C., Urgesi C., Campanella F., Eleopra R., Fabbro F. (2014). Effects of an 8-week meditation program on the implicit and explicit attitudes toward religious/spiritual self-representations. Conscious. Cogn..

[63] Crescentini C., Matiz A., Cimenti M., Pascoli E., Eleopra R., Fabbro F. (2018). Effect of mindfulness meditation on personality and psychological well-being in patients with multiple sclerosis. Int. J. MS Care.

[64] Fabbro F., Crescentini C. (2017). La meditazione orientata alla mindfulness (MOM) nella ricerca psicologica. Ricerche. Psicologia..

[65] Kabat-Zinn J. (1990). Full Catastrophe Living.

[66] Thera N. (2014). The Heart of Buddhist Meditation: The Buddha’s Way of Mindfulness.

[67] Baer R.A., Smith G.T., Hopkins J., Krietemeyer J., Toney L. (2006). Using self-report assessment methods to explore facets of mindfulness. Assessment.

[68] Giovannini C., Giromini L., Bonalume L., Tagini A., Lang M., Amadei G. (2014). The Italian five facet mindfulness questionnaire: A contribution to its validity and reliability. J. Psychopathol. Behav. Assess..

[69] Reniers R.L.E.P., Corcoran R., Drake R., Shryane N.M., Völlm B.A. (2011). The QCAE: A questionnaire of cognitive and affective empathy. J. Personal. Assess..

[70] Di Girolamo M., Giromini L., Winters C.L., Serie C.M.B., de Ruiter C. (2017). The questionnaire of cognitive and affective empathy: A comparison between paper-and-pencil versus online formats in Italian samples. J. Personal. Assess..

[71] Delvecchio G., Garzitto M., Fagnani C., Fornasari L., Stazi M.A., Picardi A., Brambilla P. (2016). Normative data and effects of age and gender on temperament and character dimensions across the lifespan in an Italian population: A cross-sectional validation study. J. Affect. Disord..

[72] Mehling W.E., Price C., Daubenmier J.J., Acree M., Bartmess E., Stewart A. (2012). The Multidimensional Assessment of Interoceptive Awareness (MAIA). PLoS ONE.

[73] Calì G., Ambrosini E., Picconi L., Mehling W.E., Committeri G. (2015). Investigating the relationship between interoceptive accuracy, interoceptive awareness, and emotional susceptibility. Front. Psychol..

[74] Ryff C.D. (1989). Happiness is everything, or is it? Explorations on the meaning of psychological well-being. J. Personal. Soc. Psychol..

[75] Sirigatti S., Stefanile C., Giannetti E., Iani L., Penzo I., Mazzeschi A. (2009). Assessment of factor structure of Ryff’s psychological well-being scales in Italian adolescents. Boll. Psicol. Appl..

[76] Viola M.M., Musso P., Inguglia C., Lo Coco A. (2016). Psychological well-being and career indecision in emerging adulthood: The moderating role of hardiness. Career Dev. Q..

[77] Zigmond A.S., Snaith R.P. (1983). The hospital anxiety and depression scale. Acta Psychiatr. Scand..

[78] Costantini M., Musso M., Viterbori P., Bonci F., Del Mastro L., Garrone O., Morasso G. (1999). Detecting psychological distress in cancer patients: Validity of the Italian version of the hospital anxiety and depression scale. Support. Care Cancer.

[79] Brennan C., Worrall-Davies A., McMillan D., Gilbody S., House A. (2010). The hospital anxiety and depression scale: A diagnostic meta-analysis of case-finding ability. J. Psychosom. Res..

[80] Bjelland I., Dahl A.A., Haug T.T., Neckelmann D. (2002). The validity of the hospital anxiety and depression scale: An updated literature review. J. Psychosom. Res..

[81] Maslach C., Jackson S.E. (1981). The measurement of experienced burnout. J. Organ. Behav..

[82] Maslach C., Jackson S.E., Schwab R.L., Maslach C., Jackson S.E., Leiter M.P. (1986). Maslach Burnout Inventory—Educators Survey (MBI-ES). MBI Study.

[83] Hartigan J.A., Wong M.A. (1979). Algorithm AS 136: A K-Means clustering algorithm. Appl. Stat..

[84] Urgesi C., Aglioti S.M., Skrap M., Fabbro F. (2010). The spiritual brain: Selective cortical lesions modulate human self-transcendence. Neuron.

[85] Ribeiro L., Atchley R.M., Oken B.S. (2017). Adherence to practice of mindfulness in novice meditators: Practices chosen, amount of time practiced, and long-term effects following a mindfulness-based intervention. Mindfulness.

[86] Forbes L., Gutierrez D., Johnson S.K. (2017). Investigating adherence to an online introductory mindfulness program. Mindfulness.

[87] Parsons C.E., Crane C., Parsons L.J., Fjorback L.O., Kuyken W. (2017). Home practice in mindfulness-based cognitive therapy and mindfulness-based stress reduction: A systematic review and meta-analysis of participants’ mindfulness practice and its association with outcomes. Behav. Res. Ther..

[88] Duan L., Zhu G. (2020). Psychological interventions for people affected by the COVID-19 epidemic. Lancet Psychiatry.

[89] Ho C.S., Chee C., Ho R. (2020). Mental health strategies to combat the psychological impact of coronavirus disease 2019 (COVID-19) beyond paranoia and panic. Ann. Acad. Med. Singapore.

[90] Fessell D., Cherniss C. (2020). Coronavirus disease 2019 (COVID-19) and beyond: Micropractices for burnout prevention and emotional wellness. J. Am. Col. Radiol..

[91] Shaw S.C. (2020). Hopelessness, helplessness and resilience: The importance of safeguarding our trainees’ mental wellbeing during the COVID-19 pandemic. Nurse Educ. Pract..

[92] Kwon C.Y., Kwak H.Y., Kim J.W. (2020). Using mind–body modalities via telemedicine during the COVID-19 crisis: Cases in the Republic of Korea. Int. J. Environ. Res. Public Health.

[93] Szcześniak D., Gładka A., Misiak B., Cyran A., Rymaszewska J. (2020). The SARS-CoV-2 and mental health: From biological mechanisms to social consequences. Prog. Neuro Psychopharmacol. Biol. Psychiatry.

[94] Behan C. (2020). The benefits of meditation and mindfulness practices during times of crisis such as Covid-19. Irish J. Psychol. Med..

[95] Polizzi C., Lynn S.J., Perry A. (2020). Stress and coping in the time of Covid-19: Pathways to resilience and recovery. Clin. Neuropsychiatry.

[96] Baiano C., Zappullo I., Conson M. (2020). Tendency to worry and fear of mental health during Italy’s COVID-19 lockdown. Int. J. Environ. Res. Public Health.

[97] Galbraith N., Boyda D., McFeeters D., Hassan T. (2020). The mental health of doctors during the Covid-19 pandemic. BJPsych Bull..

[98] Van Agteren J., Bartholomaeus J., Fassnacht D.B., Iasiello M., Ali K., Lo L., Kyrios M. (2020). Using Internet-based psychological measurement to capture the deteriorating community mental health profile during COVID-19: Observational study. JMIR Ment. Health.

[99] Vindegaard N., Benros M.E. (2020). COVID-19 pandemic and mental health consequences: Systematic review of the current evidence. Brain Behav. Immun..

[100] Luo M., Guo L., Yu M., Wang H. (2020). The psychological and mental impact of Coronavirus disease 2019 (COVID-19) on medical staff and general public—A systematic review and meta-analysis. Psychiatry Res..

[101] Lai J., Ma S., Wang Y., Cai Z., Hu J., Wei N., Tan H. (2020). Factors associated with mental health outcomes among health care workers exposed to coronavirus disease 2019. JAMA Netw. Open..

[102] Du J., Dong L., Wang T., Yuan C., Fu R., Zhang L., Bouey J. (2020). Psychological symptoms among frontline healthcare workers during COVID-19 outbreak in Wuhan. Gen. Hosp. Psychiatry.

[103] Hayes S.C., Plumb J.C. (2007). Mindfulness from the bottom up: Providing an inductive framework for understanding mindfulness processes and their application to human suffering. Psychol. Inq..

[104] Bandura A., Dienstbier R.A. (1990). Self-regulation of motivation through anticipatory and self-reactive mechanisms. Nebraska Symposium on Motivation.

[105] Connor K.M., Davidson J.R.T. (2003). Development of a new resilience scale: The Connor-Davidson Resilience Scale (CD-RISC). Depress. Anxiety.

[106] Killgore W.D.S., Taylor E.C., Cloonan S.A., Dailey N.S. (2020). Psychological resilience during the COVID-19 lockdown. Psychiatry Res..

[107] Khoury B., Lecomte T., Fortin G., Masse M., Therien P., Bouchard V., Hofmann S.G. (2013). Mindfulness-based therapy: A comprehensive meta-analysis. Clin. Psychol. Rev..

[108] Abbott R.A., Whear R., Rodgers L.R., Bethel A., Thompson C.J., Kuyken W., Dickens C. (2014). Effectiveness of mindfulness-based stress reduction and mindfulness based cognitive therapy in vascular disease: A systematic review and meta-analysis of randomised controlled trials. J. Psychosom. Res..

[109] Nam S., Toneatto T. (2016). The influence of attrition in evaluating the efficacy and effectiveness of mindfulness-based interventions. Int. J. Ment. Health Addict..

[110] Sekhon M., Cartwright M., Francis J.J. (2017). Acceptability of healthcare interventions: An overview of reviews and development of a theoretical framework. BMC Health Serv. Res..

[111] Carmody J., Reed G., Kristeller J., Merriam P. (2008). Mindfulness, spirituality, and health-related symptoms. J. Psychosom. Res..

[112] Cobb M., Puchalski C.M., Rumbold B. (2012). Oxford Textbook of Spirituality in Healthcare.

[113] Ferrell B.R., Handzo G., Picchi T., Puchalski C., Rosa W.E. (2020). The urgency of spiritual care: COVID-19 and the critical need for whole-person palliation. J. Pain Symptom Manag..

[114] Galea S., Merchant R.M., Lurie N. (2020). The mental health consequences of COVID-19 and physical distancing: The need for prevention and early intervention. JAMA Int. Med..

